# The New Mitochondrial Genome of *Hemiculterella wui* (Cypriniformes, Xenocyprididae): Sequence, Structure, and Phylogenetic Analyses

**DOI:** 10.3390/genes14122110

**Published:** 2023-11-22

**Authors:** Renyi Zhang, Tingting Zhu, Feng Yu

**Affiliations:** School of Life Sciences, Guizhou Normal University, Guiyang 550025, China; ztingting0115@163.com (T.Z.); 201711004@gznu.edu.cn (F.Y.)

**Keywords:** *Hemiculterella wui*, mitochondrial genome, phylogenetic analysis, Xenocyprididae

## Abstract

*Hemiculterella wui* is an endemic small freshwater fish, distributed in the Pearl River system and Qiantang River, China. In this study, we identified and annotated the complete mitochondrial genome sequence of *H. wui*. The mitochondrial genome was 16,619 bp in length and contained 13 protein coding genes (PCGs), two rRNA genes, 22 tRNA genes, and one control region. The nucleotide composition of the mitochondrial genome was 29.9% A, 25.3% T, 27.4% C, and 17.5% G, respectively. Most PCGs used the ATG start codon, except *COI* and *ATPase 8* started with the GTG start codon. Five PCGs used the TAA termination codon and *ATPase 8* ended with the TAG stop codon, and the remaining seven genes used two incomplete stop codons (T and TA). Most of the tRNA genes showed classical cloverleaf secondary structures, except that *tRNA^Ser(AGY)^* lacked the dihydrouracil loop. The average Ka/Ks value of the *ATPase 8* gene was the highest, while the average Ka/Ks value of the *COI* gene was the lowest. Phylogenetic analyses showed that *H. wui* has a very close relationship with *Pseudohemiculter dispar* and *H*. *sauvagei*. This study will provide a valuable basis for further studies of taxonomy and phylogenetic analyses in *H. wui* and Xenocyprididae.

## 1. Introduction

Mitochondria are semi-autonomous organelles that exist widely in eukaryotic cells and possess their genome (called the mitochondrial genome) [1]. The mitochondrial genome is a covalently closed circular double-stranded DNA molecule that can independently encode some proteins for many biological processes [2]. The mitochondrial genomes of fish usually contain 37 genes, namely 13 protein coding genes (PCGs), 2 ribosomal RNA genes (rRNAs), and 22 transfer RNA genes (tRNAs), in addition to a control region (CR) [3,4]. The CR is a non-coding region with the largest variation in the sequence and length of the entire mitochondrial genome and is generally found between the *tRNA^Pro^* and *tRNA^Phe^* genes [5]. Because the mitochondrial genome has the advantages of simple structure, small molecular weight, self-replication, strict maternal inheritance, and a fast evolution rate, it has been widely used in fish phylogeny, species identification, population genetics, adaptive evolution, etc. [5,6].

The Xenocyprididae is one of the most species-rich families of Cypriniformes, comprising approximately 160 species belonging to 45 genera [7]. *H*. *wui* (Wang, 1935) is an endemic fish that is distributed in the Pearl River system, Poyang Lake system, and the Qiantang River system, China, and is used as a small economic species in local areas. The main characteristics of *H. wui* are that absence of spinous rays in the dorsal fin and a ventral ridge from the base of the pelvic fin to the anus [8]. The common name of *H. wui* is “LanDao” in China. However, little is known about *H*. *wui*, and previous research has focused mainly on resource investigation.

In this study, we first sequenced, annotated, and characterized the complete mitochondrial genome sequence of *H. wui*. A preliminary analysis of its genetic composition and structural characteristics was conducted to provide molecular insights into the taxonomic and phylogenetic structure of the family Xenocyprididae. On this basis, combined with data from the NCBI database, the phylogenetic relationship of Xenocyprididae in this family was analyzed. Our results reveal relevant information about the mitochondrial genomes of *H. wui*, as well as the evolutionary relationships of the Xenocyprididae, which will provide a valuable basis for further studies of the evolution of *Hemiculterella* and Xenocyprididae.

## 2. Materials and Methods

### 2.1. Sample Collection, DNA Extraction, and Illumina Sequencing

The fish samples were collected from the Duliujiang River, Guizhou Province, China, and preserved in anhydrous ethanol and stored at −20 °C. Genomic DNA was extracted from the muscle of a single specimen using the DNeasy Blood & Tissue Kit (Qiagen Inc., Hilden, Germany) according to the manufacturer’s protocol. Next-generation sequencing was performed at the DNA Stories Bioinformatics Center (Chengdu, Sichuan, China). Library construction and Illumina sequencing were carried out according to Zhang et al. [9].

### 2.2. Mitochondrial Genome Assembly, Annotation, and Sequence Analysis

Mitochondrial genome assembly was performed using GetOrganelle v. 1.7.7.0 [10]. Then, MitoAnnotator 3.94 [11] was used to annotate the mitochondrial genome. The tRNA second structures were predicted using the online tool MITOS Web Server [12]. The formulae AT skew = (A − T)/(A + T) and GC skew = (G − C)/(G + C) were used to calculate the asymmetric base composition of the mitochondrial genome sequence [13]. The proportions of mitochondrial genome nucleotides and relative synonymous codon usage (RSCU) were estimated using PhyloSuite v1.2.3 [14]. The rates of non-synonymous substitutions (Ka) and synonymous substitutions (Ks) for each PCG were calculated using DnaSP 6 [15].

### 2.3. Phylogenetic Analysis

To elucidate the phylogenetic position of *H*. *wui*, we constructed phylogenetic trees using a data set of 13 PCGs of 75 species in Xenocyprididae, plus *Cyprinus carpio* Linnaeus, 1758 and *Gobiocypris rarus* Ye & Fu, 1983 as outgroups (Table 1). We used PhyloSuite v1.2.3 [13] to extract mitochondrial genes and then align the 13 PCGs using MAFFT v7.0 [16]. The optimal partition scheme and evolutionary models were determined by PartitionFinder2 [17]. The Bayesian inference (BI) phylogenetic analysis was performed in MrBayes 3.2.6 [18], employing a partition model with 2 parallel runs and 2,000,000 generations. The initial 25% of the sampled data were excluded as burn-in. Maximum likelihood (ML) phylogenetic analysis was performed in IQ-TREE v1.6.12 [19] with 5000 ultrafast bootstraps [20]. The phylogenetic trees were generated and visualized using the online tool iTOL v6 (https://itol.embl.de/) (accessed on 26 September 2023).

## 3. Results and Discussion

### 3.1. Mitochondrial Genome Organization of H. wui

**Figure 1:**
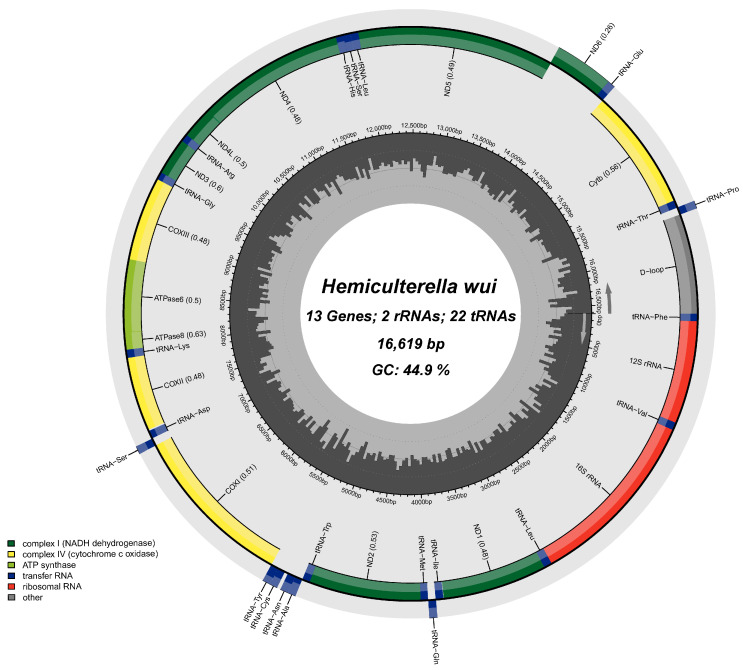
Circular map of the *H*. *wui* mitochondrial genome.

After assembly, annotation, and analysis, the mitochondrial genome (16,619 bp) of *H*. *wui* was determined in this study (GenBank Accession No.OR574832). The size of the complete mitochondrial genome of *H*. *wui* was almost the same as that of *H*. *sauvagei* Warpachowski, 1888 (16,618 bp). The mitochondrial genome of *H. wui* consisted of 13 PCGs, 2 rRNAs, 22 tRNAs, and a control region ‘D-loop’ (Figure 1; Table 2). Only 9 genes (*tRNA^Gln^*, *tRNA^Ala^*, *tRNA^Asn^*, *tRNA^Cys^*, *tRNA^Tyr^*, *tRNA^Ser (UCN)^*, *tRNA^Glu^*, *tRNA^Pro^*, and *ND6*) were encoded in the light strand, and the other genes were encoded in the heavy strand. The gene composition and order in *H. wui* were the same as in a typical fish mitochondrial genome [3,4,5].

There were 13 intergenic spacers found in the *H. wui* mitochondrial genome. The intergenic spacers varied in length from 1 bp to 32 bp (Table 2). The longest intergenic spacer was located between *tRNA^Asn^* and *tRNA^Cys^* (32 bp). Five gene overlaps were found in the *H. wui* mitochondrial genome. The minimum overlap region was located between *tRNA^Thr^* and *tRNA^Pro^* (1 bp), and the maximum overlap region was located between *ATPase 8* and *ATPase 6*, *ND4L* and *ND4* (7 bp). Mitochondrial gene overlap and gene spacers were common phenomena in teleost species [9,21].

The total nucleotide composition of the mitochondrial genome was 29.9% A, 25.3% T, 27.4% C, and 17.5% G, respectively, with a slight AT bias (55.2%) (Table 3). It was similar to other fish species in the Xenocyprididae family (Table 1). The highest A + T content was in the noncoding control region (64.4%) and the lowest A + T content was in the first codon position of PCGs (47.5%). The AT skew value was positive (0.084) in the mitochondrial genome of *H. wui*, while the GC skew value was negative (−0.22). It showed a preference for A and C bases compared to that for T and G bases.

### 3.2. Protein Coding Genes and Codon Usage

There were 13 PCGs (*ND1*, *ND2*, *ND3*, *ND4L*, *ND4*, *ND5*, *ND6*, *COI*, *COII*, *COIII*, *ATPase 6*, *ATPase 8*, and *Cyt b*) in the mitochondrial genome and their total size was 11,422 bp (Table 3). Only *ND6* was encoded on the light strand, while the rest of the PCGs were located on the heavy strand. The A + T content (54.6%) of PCGs was higher than the G + C content (45.4%). Most PCGs used the ATG start codon, but *COI* and *ATPase 8* started with GTG. The GTG start codon for *COI* was observed in many other fish species [3], but the use of GTG as the start codon in *ATPase 8* was a rare phenomenon. The termination codon TAA was used by five genes (*COI, ND1, ND4L, ND5*, and *ND6*). TAG was the stop codon of the *ATPase 8* gene. The remaining 7 genes used two incomplete stop codons (T and TA). Incomplete stop codons can be transformed into complete stop codons by post-transcriptional modification, such as polyadenylation [22].

**Figure 2:**
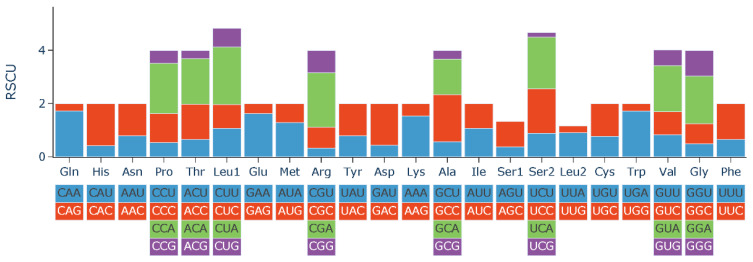
Relative synonymous codon usage (RSCU) of the mitochondrial genome for *H*. *wui*.

The RSCU value was a measure of codon usage preference in the genome. The number of codons and the RSCU of 13 PCGs are presented in Table 4 and Figure 2. A total of 3804 amino acid triplets were used in the 13 PCGs. The most commonly used amino acids were Leu, followed by Ala, Thr, Ile, and Gly. The least commonly used amino acid was Cys. The most commonly used codon was CUA, followed by UUC, AUU, and GCC. The least frequent codon was CGU, not including stop codons.

### 3.3. Ribosomal and Transfer RNA Genes

The mitochondrial genome of *H. wui* has two rRNAs (12S rRNA and 16S rRNA), both encoded in the heavy stand. They were close together in the genome, separated by a single tRNA. The 12S rRNA was located between *tRNA^Phe^* and *tRNA^Val^* with a length of 964 bp. The 16S rRNA was located between *tRNA^Val^* and *tRNA^Leu (UUR)^* with a length of 1690 bp. The A + T content of the two rRNA was 53.70%. The two rRNA genes had a positive AT skew value of 0.245 and a negative GC skew value of −0.046 (Table 3).

**Figure 3:**
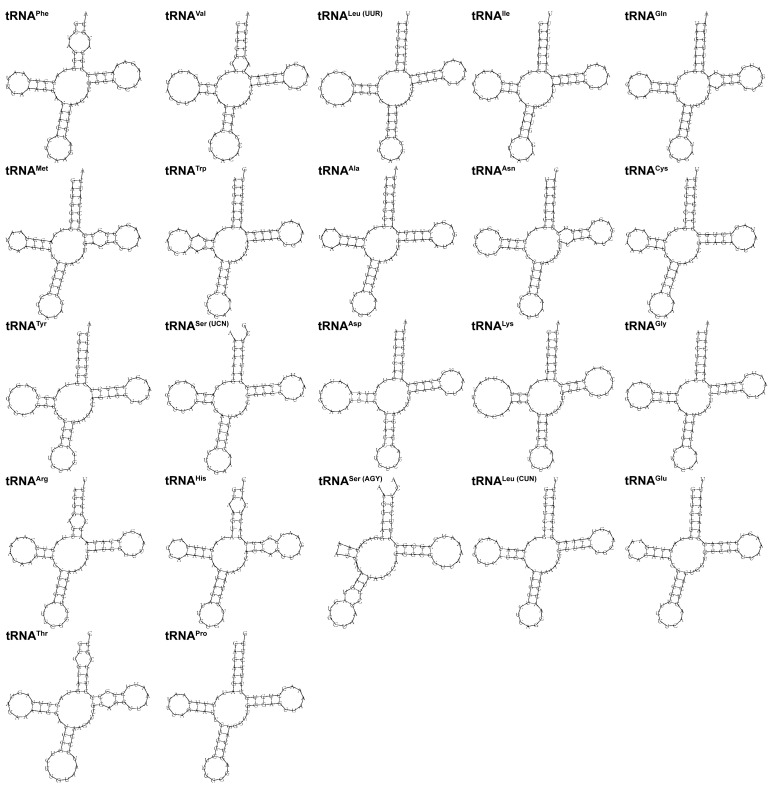
Predicted secondary structures of 22 tRNAs in the mitochondrial genome of *H*. *wui*.

Transfer RNA, also known as transfer ribonucleic acid, is often referred to as tRNA. As shown in Figure 3, we found that most tRNA genes had a typical cloverleaf secondary structure, except *tRNA^Ser(AGY)^* lacked the dihydrouracil loop (DHU loop). The tRNA genes varied in length from 68 bp (*tRNA^Cys^*) to 76 bp (*tRNA^Leu (UUR)^* and *tRNA^Lys^*) and were scattered across the mitochondrial genome. The sum of the A + T content of all tRNAs was 55.8%. It showed a preference for AT bases. The AT skew and GC skew values were 0.029 and 0.056, respectively.

### 3.4. Control Region

The control region, also known as the AT rich region or D-loop, was 934 bp in length. It was located between *tRNA^Pro^* and *tRNA^Phe^*, similar to a typical fish mitochondrial genome [3]. The nucleotide composition was 32.9% A, 31.5% T, 14.9% G, and 20.8% C (Table 3). This region had the highest AT content in the entire mitochondrial genome.

### 3.5. Selection Analysis

**Figure 4:**
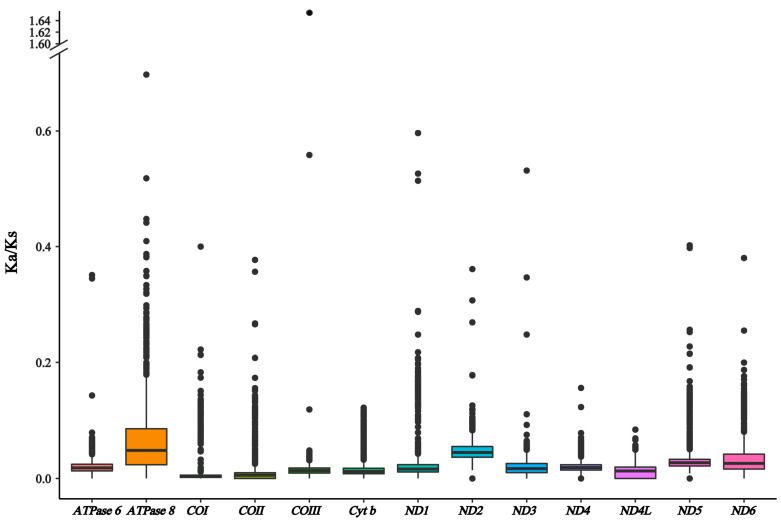
Ka/Ks values for 13 PCGs from 75 mitochondrial genomes of Xenocyprididae.

Among the 13 PCGs of the Xenocyprididae, the average value of Ka/Ks of *ATPase 8* was the highest, while the average value of Ka/Ks of *COI* was the lowest (Figure 4). It implies that *ATPase 8* might evolve more rapidly than other mitochondrial protein coding genes. Even under different selection pressures, the evolutionary patterns of 13 PCGs in Xenocyprididae were similar to those of *Sinocyclocheilus* (Fang, 1936) fishes [23] and Labeoninae [9]. Furthermore, the average Ka/Ks ratios of all PCGs were much lower than one, indicating that these genes were all under a strong purifying selection.

### 3.6. Phylogenetic Analysis

**Figure 5:**
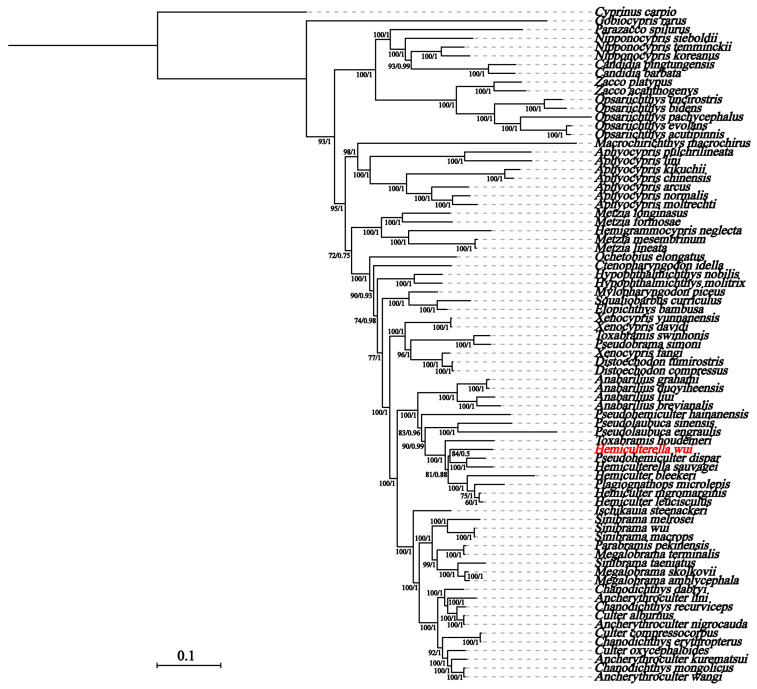
The phylogeny of *H. wui* with other species of Xenocyprididae based on the concatenated nucleotide sequences of 13 PCGs. The Maximum likelihood bootstrap values and Bayesian posterior probabilities are superimposed on each node.

We used a total of 75 species of Xenocyprididae to construct phylogenetic trees based on 13 PCGs in the mitochondrial genome, with *C. carpio* and *G. rarus* as outgroups. The two methods used to build the phylogenetic tree yielded similar topological structures (Figure 5). The phylogenetic trees showed that *H. wui, P*. *dispar* (Peters, 1881), and *H*. *sauvagei,* were clustered into a group (ML bootstrap value = 84%, Bayesian posterior probability = 0.5). The differences in external morphology between *Pseudohemiculter* Nichols & Pope, 1927 and *Hemiculterella* Warpachowski, 1888 are not obvious. The main difference is that the last unbranched dorsal fin ray of *Pseudohemiculter* is hard, whereas that of *Hemiculterella* is soft [7]. The monophyly of *Hemiculterella*, *Pseudohemiculter*, and *Hemiculter* was not supported. More extensive sampling and multilocus markers are required to understand the robust phylogenetic relationships of *Pseudohemiculter*, *Hemiculterella*, *Hemiculter*, and related genera.

## 4. Conclusions

In conclusion, we analyzed and described the characterization of the mitochondrial genome of *H. wui* and the phylogenetic relationship position in the Xenocyprididae, which will provide a valuable basis for further studies of *H*. *wui* and the evolutionary relationship of Xenocyprididae.

## Figures and Tables

**Table 1:** Species information for phylogenetic analysis.

No.	Species	GenBank Accession No.	Size (bp)	A%	T%	G%	C%	A + T%	AT Skew	GC Skew
1	*Anabarilius brevianalis* Zhou & Cui, 1992	MK757491.1	16,610	28.3	24.6	18.7	28.4	52.9	0.070	−0.205
2	*Anabarilius duoyiheensis* Li, Mao & Lu, 2002	NC_068241.1	16,614	28.9	24.9	18.4	27.9	53.8	0.074	−0.205
3	*Anabarilius grahami* (Regan, 1908)	MF370204.1	16,612	28.9	24.8	18.3	27.9	53.7	0.077	−0.208
4	*Anabarilius liui* (Chang, 1944)	MG702493.1	16,608	28.5	24.4	18.5	28.5	52.9	0.077	−0.212
5	*Ancherythroculter kurematsui* (Kimura, 1934)	NC_029707.1	16,621	31.2	24.9	16.2	27.7	56.1	0.112	−0.263
6	*Ancherythroculter lini* Luo, 1994	NC_027741.1	16,616	30.8	24.7	16.5	28.0	55.5	0.109	−0.259
7	*Ancherythroculter nigrocauda* Yih & Wu, 1964	NC_021414.1	16,623	31.2	24.8	16.2	27.8	56.0	0.114	−0.262
8	*Ancherythroculter wangi* (Tchang, 1932)	NC_037405.1	16,622	31.2	24.8	16.2	27.8	56.0	0.113	−0.264
9	*Aphyocypris arcus* (Lin, 1931)	NC_015540.1	16,617	31.4	27.1	15.7	25.7	58.5	0.074	−0.241
10	*Aphyocypris chinensis* Günther, 1868	NC_008650.1	16,606	30.7	27.7	16.5	25.1	58.4	0.053	−0.207
11	*Aphyocypris kikuchii* (Oshima, 1919)	NC_019620.1	16,601	30.8	27.7	16.5	25.0	58.5	0.052	−0.206
12	*Aphyocypris lini* (Weitzman & Chan, 1966)	NC_062821.1	16,613	30.4	26.9	16.6	26.0	57.3	0.061	−0.221
13	*Aphyocypris moltrechti* (Regan, 1908)	NC_019621.1	16,617	31.2	27.4	16.0	25.5	58.6	0.066	−0.229
14	*Aphyocypris normalis* Nichols & Pope, 1927	NC_015538.1	16,619	31.2	27.0	15.9	25.8	58.2	0.073	−0.237
15	*Aphyocypris pulchrilineata* Zhu, Zhao & Huang, 2013	MK387702.1	16,610	30.5	26.6	16.6	26.2	57.1	0.069	−0.224
16	*Candidia barbata* (Regan, 1908)	NC_037156.1	16,608	30.3	26.9	16.7	26.1	57.2	0.059	−0.219
17	*Candidia pingtungensis* Chen, Wu & Hsu, 2008	NC_028596.1	16,612	29.9	26.9	17.1	26.2	56.8	0.053	−0.211
18	*Chanodichthys dabryi* (Bleeker, 1871)	NC_021418.1	16,622	31.5	25.0	15.9	27.6	56.5	0.116	−0.268
19	*Chanodichthys erythropterus* (Basilewsky, 1855)	MN105126.1	16,623	31.0	25.1	16.3	27.6	56.1	0.106	−0.255
20	*Chanodichthys mongolicus* (Basilewsky, 1855)	NC_008683.1	16,622	31.2	24.9	16.2	27.8	56.1	0.113	−0.264
21	*Chanodichthys recurviceps* (Richardson, 1846)	NC_024277.1	16,622	31.4	24.8	16.1	27.8	56.2	0.117	−0.266
22	*Ctenopharyngodon idella* (Valenciennes, 1844)	NC_010288.1	16,609	31.9	26.2	15.6	26.3	58.1	0.098	−0.253
23	*Culter alburnus* Basilewsky, 1855	NC_013616.1	16,622	31.2	24.8	16.2	27.8	56.0	0.114	−0.262
24	*Culter compressocorpus* (Yih & Chu, 1959)	NC_024183.1	16,623	31.1	25.1	16.3	27.5	56.2	0.105	−0.254
25	*Culter oxycephaloides* Kreyenberg & Pappenheim, 1908	KY404014.1	16,619	31.3	24.8	16.1	27.9	56.1	0.117	−0.269
26	*C*. *carpio* Linnaeus, 1758	NC_018035.1	16581	31.9	24.8	15.7	27.5	56.7	0.125	−0.272
27	*Distoechodon compressus* (Nichols, 1925)	NC_067894.1	16,621	31.4	25.4	16.0	27.2	56.8	0.107	−0.260
28	*Distoechodon tumirostris* Peters, 1881	NC_011208.1	16,620	31.4	25.4	16.0	27.2	56.8	0.106	−0.261
29	*Elopichthys bambusa* (Richardson, 1845)	NC_024834.1	16,619	30.1	27.2	16.9	25.8	57.3	0.049	−0.209
30	*G*. *rarus* Ye & Fu, 1983	NC_018099.1	16,601	29.5	27.6	17.2	25.7	57.1	0.034	−0.198
31	*Hemiculter bleekeri* Warpachowski, 1888	NC_029831.1	16,615	29.3	25.6	17.8	27.3	54.9	0.067	−0.211
32	*Hemiculter leucisculus* (Basilewsky, 1855)	NC_022929.1	16,617	30.4	25.4	17.1	27.2	55.8	0.090	−0.228
33	*Hemiculter nigromarginis* Fang, 1942	NC_036740.1	16,621	30.4	25.4	17.1	27.1	55.8	0.089	−0.228
34	*Hemiculterella sauvagei* Warpachowski, 1888	NC_026693.1	16,618	29.9	25.6	17.4	27.0	55.5	0.078	−0.216
35	*H*. *wui* (Wang, 1935)	OR574832	16,619	29.9	25.3	17.5	27.4	55.2	0.084	−0.22
36	*Hemigrammocypris neglecta* (Stieler, 1907)	NC_015548.1	16,615	31.2	26.3	16.7	25.8	57.5	0.085	−0.215
37	*Hypophthalmichthys molitrix* (Valenciennes, 1844)	NC_010156.1	16,620	31.8	25.6	15.8	26.9	57.4	0.109	−0.261
38	*Hypophthalmichthys nobilis* (Richardson, 1845)	NC_010194.1	16,621	31.6	25.3	15.9	27.1	56.9	0.111	−0.259
39	*Ischikauia steenackeri* (Sauvage, 1883)	NC_008667.1	16,620	31.4	25.1	16.1	27.4	56.5	0.111	−0.261
40	*Macrochirichthys macrochirus* (Valenciennes, 1844)	NC_015551.1	16857	32.5	26.1	15.2	26.2	58.6	0.108	−0.266
41	*Megalobrama amblycephala* Yih, 1955	NC_010341.1	16,623	31.2	24.7	16.2	27.9	55.9	0.117	−0.265
42	*Megalobrama skolkovii* (Basilewsky, 1855)	NC_024422.1	16,620	31.2	24.7	16.2	27.9	55.9	0.116	−0.266
43	*Megalobrama terminalis* (Richardson, 1846)	NC_018816.1	16,623	31.1	24.8	16.3	27.8	55.9	0.113	−0.26
44	*Metzia formosae* (Oshima, 1920)	NC_022458.1	16,614	31.4	25.6	16.2	26.8	57.0	0.102	−0.246
45	*Metzia lineata* (Pellegrin, 1907)	NC_031541.1	16,614	32.1	26.9	15.6	25.4	59.0	0.089	−0.241
46	*Metzia longinasus* Gan, Lan & Zhang, 2009	NC_024729.1	16,614	31.9	26.2	15.7	26.2	58.1	0.098	−0.251
47	*Metzia mesembrinum* (Jordan & Evermann, 1902)	NC_023797.1	16,611	32.0	26.8	15.7	25.5	58.8	0.088	−0.238
48	*Mylopharyngodon piceus* (Richardson, 1846)	NC_011141.1	16,609	32.0	24.5	15.7	27.8	56.5	0.132	−0.278
49	*Nipponocypris koreanus* (Kim, Oh & Hosoya, 2005)	NC_025286.1	16,615	30.0	26.8	17.1	26.1	56.8	0.056	−0.209
50	*Nipponocypris sieboldii* (Temminck & Schlegel, 1846)	NC_008653.1	16,616	30.1	25.8	16.9	27.2	55.9	0.078	−0.233
51	*Nipponocypris temminckii* (Temminck & Schlegel, 1846)	NC_027664.1	16,615	30.3	26.5	16.8	26.3	56.8	0.067	−0.221
52	*Ochetobius elongatus* (Kner, 1867)	NC_025646.1	16,613	31.0	25.4	16.3	27.4	56.4	0.099	−0.255
53	*Opsariichthys acutipinnis* (Bleeker, 1871)	NC_028595.1	16,615	28.2	26.6	18.0	27.2	54.8	0.030	−0.204
54	*Opsariichthys bidens* Günther, 1873	NC_008744.1	16,611	27.2	26.7	19.1	27.1	53.9	0.010	−0.174
55	*Opsariichthys evolans* (Jordan & Evermann, 1902)	NC_033948.1	16,616	28.2	26.6	18.0	27.2	54.8	0.029	−0.203
56	*Opsariichthys pachycephalus* Günther, 1868	NC_033949.1	16,606	27.9	26.8	18.3	27.1	54.7	0.020	−0.194
57	*Opsariichthys uncirostris* (Temminck & Schlegel, 1846)	NC_008652.1	16,613	27.2	26.7	18.9	27.2	53.9	0.008	−0.181
58	*Parabramis pekinensis* (Basilewsky, 1855)	NC_022678.1	16,622	31.1	24.8	16.3	27.8	55.9	0.113	−0.261
59	*Parazacco spilurus* (Günther, 1868)	NC_023786.1	16,612	30.4	26.9	16.6	26.0	57.3	0.062	−0.220
60	*Plagiognathops microlepis* (Bleeker, 1871)	NC_022711.1	16,623	30.6	25.2	16.9	27.3	55.8	0.097	−0.236
61	*Pseudobrama simoni* (Bleeker, 1864)	NC_022852.1	16,618	31.6	27.2	15.7	25.5	58.8	0.075	−0.238
62	*P*. *dispar* (Peters, 1881)	NC_020435.1	16,620	30.3	25.5	17.1	27.1	55.8	0.086	−0.224
63	*Pseudohemiculter hainanensis* (Boulenger, 1900)	NC_065693.1	16,647	29.7	24.8	17.5	28.0	54.5	0.089	−0.230
64	*Pseudolaubuca engraulis* (Nichols, 1925)	NC_020462.1	16,612	27.1	25.9	19.5	27.5	53.0	0.023	−0.171
65	*Pseudolaubuca sinensis* Bleeker, 1864	NC_026712.1	16,617	29.6	25.8	17.6	26.9	55.4	0.069	−0.209
66	*Sinibrama macrops* (Günther, 1868)	NC_020013.1	16,626	30.7	24.7	16.6	27.9	55.4	0.110	−0.253
67	*Sinibrama melrosei* (Nichols & Pope, 1927)	NC_063731.1	16,619	30.7	25.5	16.6	27.2	56.2	0.092	−0.242
68	*Sinibrama taeniatus* (Nichols, 1941)	NC_026119.1	16,623	31.3	25.8	16.1	26.8	57.1	0.097	−0.248
69	*Sinibrama wui* (Rendahl, 1933)	NC_068747.1	16,626	30.7	24.6	16.7	28.0	55.3	0.110	−0.253
70	*Squaliobarbus curriculus* (Richardson, 1846)	NC_019652.1	16,619	31.2	25.0	16.1	27.7	56.2	0.111	−0.264
71	*Toxabramis houdemeri* Pellegrin, 1932	NC_029348.1	16,618	30.8	25.3	16.6	27.3	56.1	0.098	−0.243
72	*Toxabramis swinhonis* Günther, 1873	NC_029249.1	16,622	31.2	26.5	16.1	26.1	57.7	0.081	−0.237
73	*Xenocypris davidi* Bleeker, 1871	NC_013072.1	16,630	31.3	25.4	16.2	27.2	56.7	0.104	−0.254
74	*Xenocypris fangi* Tchang, 1930	NC_056130.1	16,619	31.2	25.3	16.2	27.3	56.5	0.105	−0.255
75	*Xenocypris yunnanensis* Nichols, 1925	NC_035954.1	16,630	31.3	25.4	16.1	27.2	56.7	0.104	−0.255
76	*Zacco acanthogenys* (Bleeker, 1871)	NC_028546.1	16,611	29.1	27.2	17.6	26.0	56.3	0.033	−0.192
77	*Zacco platypus* (Temminck & Schlegel, 1846)	NC_023105.1	16,611	29.0	27.2	17.8	26.1	56.2	0.032	−0.190

**Table 2:** Organization and characterization of the *H*. *wui* mitochondrial genome.

Gene	Strand	Location (bp)	Size (bp)	Intergenic Nucleotide	Anticodon	Start/Stop Codons
*tRNA^Phe^*	H	1–69	69	0	GAA	
*12S rRNA*	H	70–1033	964	0		
*tRNA^Val^*	H	1034–1105	72	0	TAC	
*16S rRNA*	H	1106–2795	1690	0		
*tRNA^Leu (UUR)^*	H	2796–2871	76	1	TAA	
*ND1*	H	2873–3847	975	4		ATG/TAA
*tRNA^Ile^*	H	3852–3923	72	−2	GAT	
*tRNA^Gln^*	L	3922–3992	71	1	TTG	
*tRNA^Met^*	H	3994–4062	69	0	CAT	
*ND2*	H	4063–5107	1045	0		ATG/T
*tRNA^Trp^*	H	5108–5178	71	1	TCA	
*tRNA^Ala^*	L	5180–5248	69	1	TGC	
*tRNA^Asn^*	L	5250–5322	73	32	GTT	
*tRNA^Cys^*	L	5355–5422	68	1	GCA	
*tRNA^Tyr^*	L	5424–5494	71	1	GTA	
*COI*	H	5496–7046	1551	0		GTG/TAA
*tRNA^Ser (UCN)^*	L	7047–7117	71	3	TGA	
*tRNA^Asp^*	H	7121–7194	74	13	GTC	
*COII*	H	7208–7898	691	0		ATG/T
*tRNA^Lys^*	H	7899–7974	76	1	TTT	
*ATPase 8*	H	7976–8140	165	−7		GTG/TAG
*ATPase 6*	H	8134–8816	683	0		ATG/TA
*COIII*	H	8817–9601	785	0		ATG/TA
*tRNA^Gly^*	H	9602–9673	72	0	TCC	
*ND3*	H	9674–10,022	349	0		ATG/T
*tRNA^Arg^*	H	10,023–10,092	70	0	TCG	
*ND4L*	H	10,093–10,389	297	−7		ATG/TAA
*ND4*	H	10,383–11,764	1382	0		ATG/TA
*tRNA^His^*	H	11,765–11,833	69	0	GTG	
*tRNA^Ser^ (AGY)*	H	11,834–11,902	69	1	GCT	
*tRNA^Leu^ (CUN)*	H	11,904–11,976	73	0	TAG	
*ND5*	H	11,977–13,812	1836	−4		ATG/TAA
*ND6*	L	13,809–14,330	522	0		ATG/TAA
*tRNA^Glu^*	L	14,331–14,399	69	4	TTC	
*Cyt b*	H	14,404–15,544	1141	0		ATG/T
*tRNA^Thr^*	H	15,545–15,616	72	−1	TGT	
*tRNA^Pro^*	L	15,616–15,685	70	0	TGG	
*Control region*	H	15,686–16,619	934	0		

**Table 3:** List of the nucleotide composition, AT skew, and GC skew of the *H. wui* mitochondrial genome.

Regions	Size (bp)	A%	T%	G%	C%	A + T%	G + C%	AT Skew	GC Skew
Full genome	16,619	29.9	25.3	17.5	27.4	55.2	44.9	0.084	−0.22
PCGs	11,422	27.6	27.0	17.1	28.3	54.6	45.4	0.011	−0.248
1st codon position	3804	26.5	21.0	26.4	26.1	47.5	52.5	0.116	0.005
2nd codon position	3804	18.5	40.5	13.7	27.3	59.0	41.0	−0.374	−0.333
3rd codon position	3804	37.9	19.4	11.1	31.6	57.3	42.7	0.322	−0.478
*ATPase 6*	683	27.1	29.6	14.5	28.8	56.7	43.3	−0.044	−0.331
*ATPase 8*	165	35.8	23.6	12.1	28.5	59.4	40.6	0.204	−0.403
*COI*	1551	26.6	28.2	18.6	26.6	54.8	45.2	−0.028	−0.178
*COII*	691	29.8	26.0	16.6	27.5	55.8	44.1	0.067	−0.246
*COIII*	785	27.3	27.6	17.1	28.0	54.9	45.1	−0.007	−0.243
*Cyt b*	1141	28.0	26.2	15.9	30.0	54.2	45.9	0.032	−0.308
*ND1*	975	26.8	24.9	17.5	30.8	51.7	48.3	0.036	−0.274
*ND2*	1045	28.3	22.9	17.2	31.6	51.2	48.8	0.107	−0.294
*ND3*	349	25.8	28.7	17.2	28.4	54.5	45.6	−0.053	−0.245
*ND4L*	1382	29.1	27.0	15.9	28.0	56.1	43.9	0.037	−0.275
*ND4*	297	25.9	25.3	16.5	32.3	51.2	48.8	0.013	−0.324
*ND5*	1836	30.4	26.0	14.4	29.2	56.4	43.6	0.077	−0.34
*ND6*	522	14.0	39.1	32.0	14.9	53.1	46.9	−0.473	0.363
rRNAs	2654	33.4	20.3	22.1	24.2	53.7	46.3	0.245	−0.046
tRNAs	1566	28.7	27.1	23.3	20.8	55.8	44.1	0.029	0.056
Control region	934	32.9	31.5	14.9	20.8	64.4	35.7	0.022	−0.165

**Table 4:** Codon number in the *H. wui* mitochondrial PCGs.

Amino Acid	Codon	Count	RSCU	Amino Acid	Codon	Count	RSCU
Phe (F)	UUU	74	0.66	Tyr (Y)	UAU	45	0.79
	UUC	151	1.34		UAC	69	1.21
Leu (L)	UUA	94	0.91	stop codon	UAA	5	3.33
	UUG	26	0.25		UAG	1	0.67
	CUU	111	1.07	His (H)	CAU	22	0.42
	CUC	92	0.89		CAC	82	1.58
	CUA	224	2.16	Gln (Q)	CAA	84	1.73
	CUG	74	0.71		CAG	13	0.27
Ile (I)	AUU	149	1.06	Asn (N)	AAU	48	0.79
	AUC	132	0.94		AAC	73	1.21
Met (M)	AUA	112	1.29	Lys (K)	AAA	61	1.54
	AUG	61	0.71		AAG	18	0.46
Val (V)	GUU	50	0.83	Asp (D)	GAU	17	0.44
	GUC	52	0.86		GAC	61	1.56
	GUA	105	1.74	Glu (E)	GAA	84	1.63
	GUG	35	0.58		GAG	19	0.37
Ser (S)	UCU	35	0.88	Cys (C)	UGU	10	0.77
	UCC	67	1.68		UGC	16	1.23
	UCA	77	1.93	Trp (W)	UGA	104	1.72
	UCG	7	0.18		UGG	17	0.28
Pro (P)	CCU	29	0.54	Arg (R)	CGU	6	0.32
	CCC	59	1.09		CGC	15	0.79
	CCA	102	1.89		CGA	39	2.05
	CCG	26	0.48		CGG	16	0.84
Thr (T)	ACU	50	0.66	Ser (S)	AGU	15	0.38
	ACC	99	1.32		AGC	38	0.95
	ACA	128	1.7	stop codon	AGA	0	0
	ACG	24	0.32		AGG	0	0
Ala (A)	GCU	48	0.57	Gly (G)	GGU	30	0.49
	GCC	149	1.77		GGC	46	0.75
	GCA	112	1.33		GGA	109	1.79
	GCG	28	0.33		GGG	59	0.97

## Data Availability

Mitochondrial genome sequence data supporting the findings of this study are openly available from the GenBank of the National Center for Biotechnology Information (NCBI) at https://www.ncbi.nlm.nih.gov (accession number: OR574832) accessed on 18 October 2023.
